# Redetermination of the crystal structure of yttrium chromium tetra­boride, YCrB_4_, from single-crystal X-ray diffraction data

**DOI:** 10.1107/S2056989023008952

**Published:** 2023-10-26

**Authors:** Makoto Tokuda, Kunio Yubuta, Toetsu Shishido, Kazumasa Sugiyama

**Affiliations:** a Institute of Industrial Nano Materials, Kumamoto University, 2-39-1 Kurokami, Chuo-ku, Kumamoto 860-8555, Japan; b Department of Applied Quantum Physics and Nuclear Engineering, Kyushu University, Fukuoka 819-0395, Japan; c Institute for Materials Research, Tohoku University, 2-1-1 Katahira, Aoba-ku, Sendai 980-8577, Japan; University of Kentucky, USA

**Keywords:** single-crystal diffraction, crystal structure, boride

## Abstract

The structural parameters of yttrium chromium tetra­boride YCrB_4_ were refined based on single-crystal X-ray diffraction data. YCrB_4_ is ortho­rhom­bic, having a space group of type *Pbam* (No. 55) and with lattice parameters of *a* = 5.9425 (2), *b* = 11.4831 (4), *c* = 3.4643 (1) Å. The Y and Cr atoms are located at Wyckoff 4*h* sites (*x*, *y*, 0) and B atoms at the Wyckoff 4*g* sites (*x*, *y*, 1/2). The first structural investigation of YCrB_4_ was performed using a single crystalline sample [Kuz’ma, (1970 ▸). *Kristallografiya*. **15**, 372–374]. The present study successfully refined all the positional and atomic displacement parameters of the Y, Cr, and B atoms.

## Abstract 2

The structural parameters of yttrium chromium tetra­boride YCrB_4_ were refined based on single-crystal X-ray diffraction data. The present study successfully refined all the positional and atomic displacement parameters of the Y, Cr, and B atoms.

## 1. Chemical context

The investigation of AlB_2_-type-analogous inter­metallic compounds (*R*B_2_, *M*B_2_, *RM*B_4_, and *R*_2_ *M*B_6_; *R* = rare-earth, *M* = aluminium or transition metal) has been pursued using various experimental and theoretical methods. Recently, hydrogenated monolayer boron sheets (borophene) have attracted attention because of their topological Dirac nodal loops, with the AlB_2_-type-analogous compounds expected to be the parent materials (Cuong *et al.*, 2020 ▸; Niibe *et al.*, 2021 ▸; Tateishi *et al.*, 2022 ▸). YCrB_4_ is a parent material candidate for the synthesis of (5-7)-*α*-borophene sheets (*i.e.*, boron networks with five- and seven-membered rings), for which detailed structural data are currently required (Zhang *et al.*, 2022 ▸, 2023 ▸). YCrB_4_ is a promising semiconductor material with good thermoelectric properties and a good power factor of 6.0 µW cm^−1^ K^−2^ at 500 K (Simonson & Poon, 2010 ▸; Flipo *et al.*, 2021 ▸). The YCrB_4_ compound is also expected to be a super-hard material (Akopov *et al.*, 2018 ▸) and a narrow bandgap semiconductor (Medvedeva *et al.*, 2002 ▸). A theoret­ically calculated Debye temperature *θ*_D_ for YCrB_4_ of 965 K was given by Candan *et al.* (2019 ▸). Notably, Kuz’ma (1970 ▸) conducted the first structural analysis of YCrB_4_.

## 2. Structural commentary

**Figure 1:**
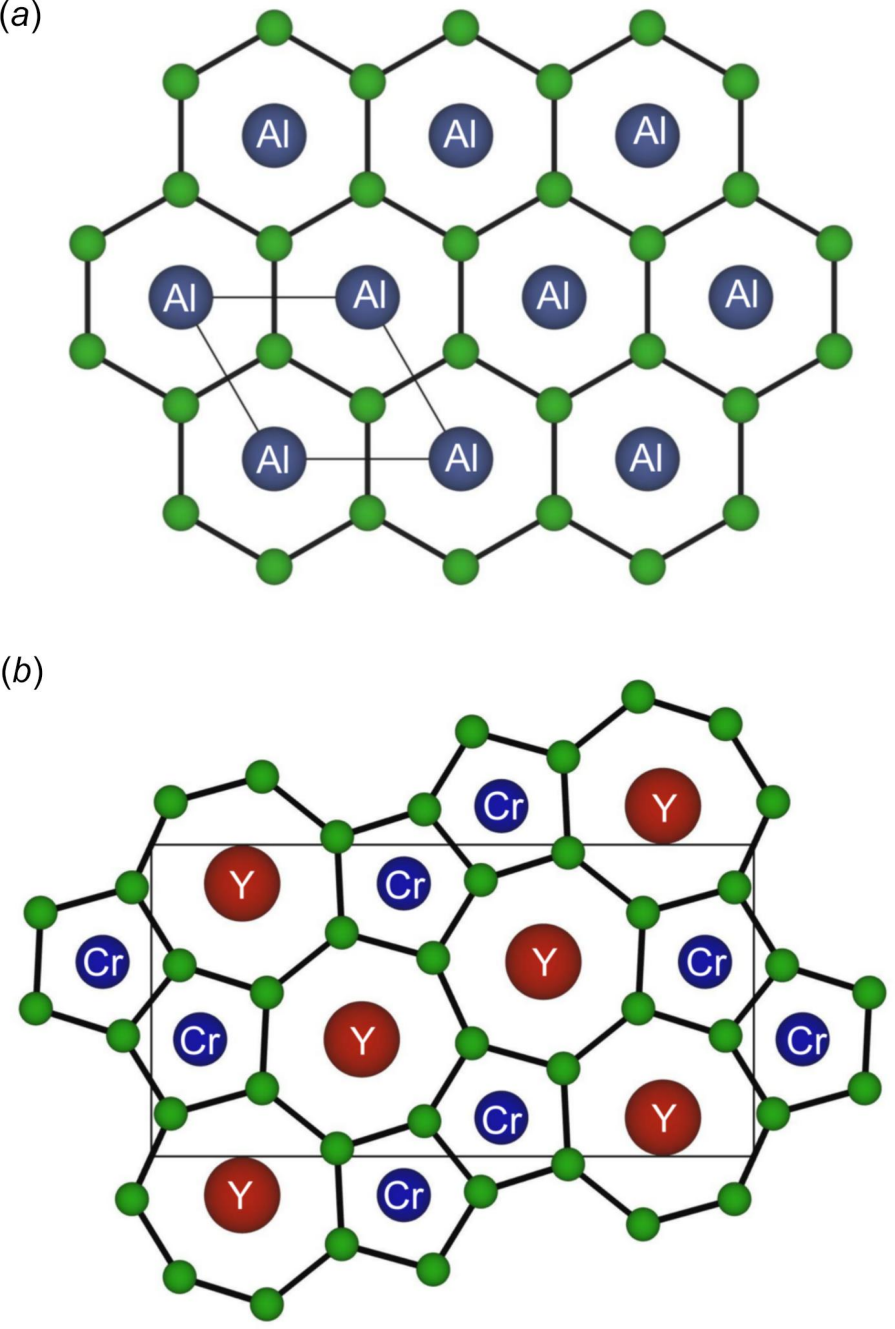
Crystal structure of boron-metal-layered compounds. Honeycomb borophene layer in AlB_2_ (*b*) and (5–7)-*α*-borophene layer in YCrB_4_ (*a*) viewed along the *c*-axis and illustrated using *VESTA* (Momma & Izumi, 2011 ▸).

**Figure 2:**
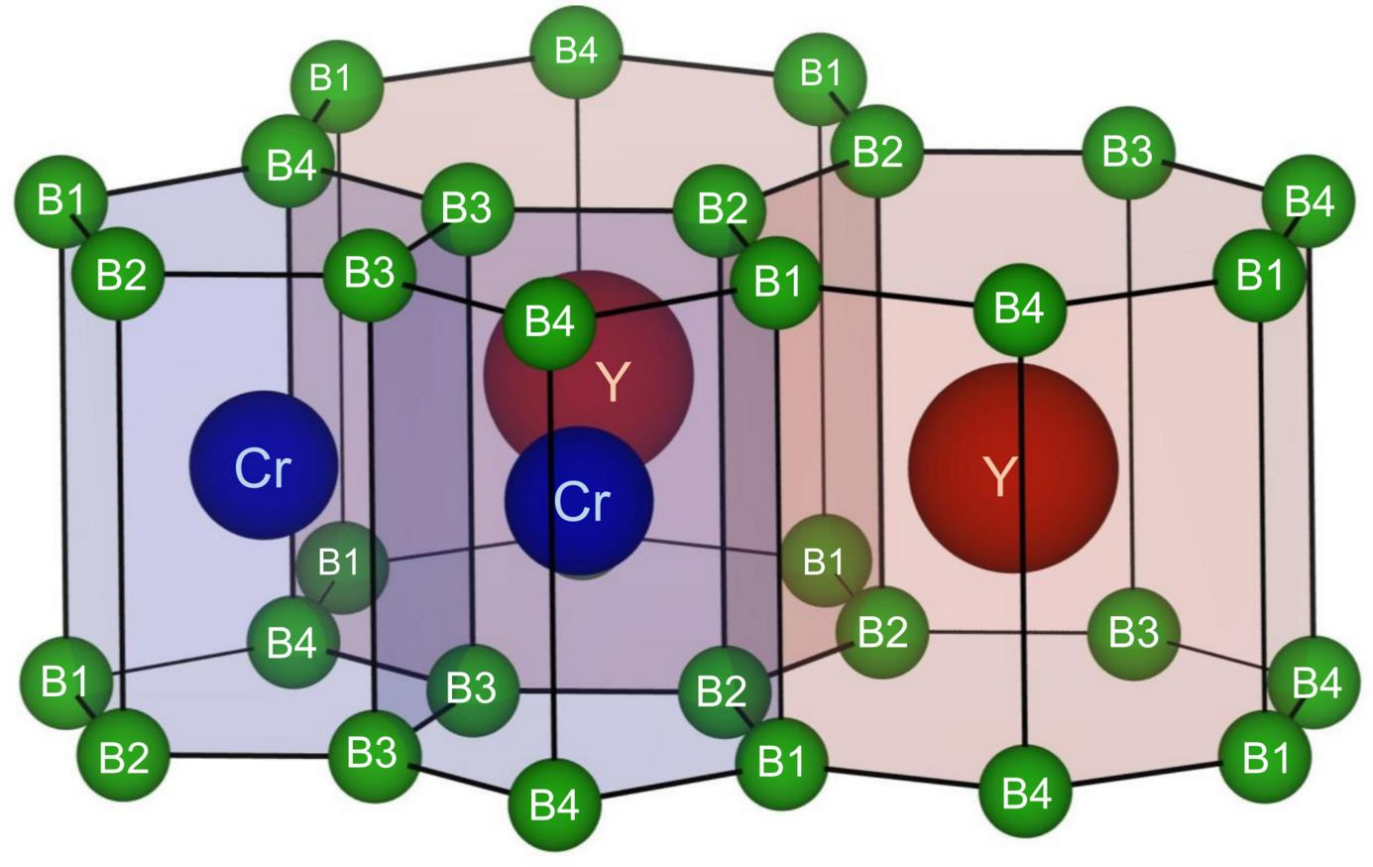
Cr and Y atoms settle in the center of the penta­gonal CrB_10_ and hepta­gonal YB_14_ prisms, respectively.

The AlB_2_-type-analogous compounds are composed of borophene layers stacked with other metal atomic layers. The boron network is composed of six-membered rings (honeycomb layer), with *R* or *M* atoms located at the center of a hexa­gonal prism formed by twelve boron atoms (Fig. 1 ▸ *a*). YCrB_4_ exhibits an ordered and rearranged crystal structure derived from the AlB_2_-type structure of YB_2_ or CrB_2_ (Kuz’ma, 1970 ▸). The six-membered rings are rearranged into five- and seven-membered rings [(5-7)-*α*-borophene layer] due to the ordering of Cr and Y (Fig. 1 ▸ *b*). These (5-7)-*α*-borophene layers are accumulated along the *c* axis in an *αα* stacking sequence with the metal atomic layers. The Cr and Y atoms are located at the centers of the CrB_10_ penta­gonal and YB_14_ hepta­gonal prisms, respectively (Fig. 2 ▸). With different arrangements of the *M*B_10_ penta­gonal prism and *R*B_14_ hepta­gonal prism, three distinct structural types have been reported for the AlB_2_-type-analogous compounds: (5-7)-*α*-type (YCrB_4_-type: Rogl, 1978 ▸; Sobczak & Rogl, 1979 ▸), (5-7)-*β*-type (ThMoB_4_-type: Rogl & Nowotny 1974 ▸; Veremchuk *et al.*, 2008 ▸), and (5-6-7)-*γ*-type (Y_2_ReB_6_-type: Kuz’ma & Svarichevskaya, 1972 ▸; Okada *et al.*, 2006 ▸).

The Cr—B and Y—B inter­atomic distances are in the range of 2.2677 (15)–2.3254 (10) and 2.6177 (16)–2.7478 (14) Å, respectively (Table 1 ▸), which are close to the sums of the respective Goldschmidt radii (*r*_B_ = 0.97 Å, *r*_Cr_ = 1.36 Å, and *r*_Y_ = 1.81 Å; Brandes & Brook, 1992 ▸). The inter­planar Cr—Cr distance is 2.3745 (4) Å, indicating a strong correlation between the Cr atoms. The intra- and inter­planar Y—Y distances are 3.7446 (3)–3.7653 (5) and 3.46425 (12) Å, respectively (the latter simply corresponding to the *c*-axis length). The inter­planar Y—Y distance is much smaller than the sum of radii of the Y atoms. A short intra­planar *R—R* distance can be observed in various *R*–*M*–B systems with layered structures (Higashi *et al.*, 1988 ▸; Tokuda *et al.*, 2022 ▸). The B—B inter­atomic distances within the penta­gons and hepta­gons in YCrB_4_ are in the ranges 1.724 (4)–1.828 (2) and 1.741 (3)–1.832 (2) Å, respectively, similar to the average B—B covalent bonding distances of 1.77 Å in *α*-rhombohedral boron (Donohue, 1974 ▸).

**Figure 3:**
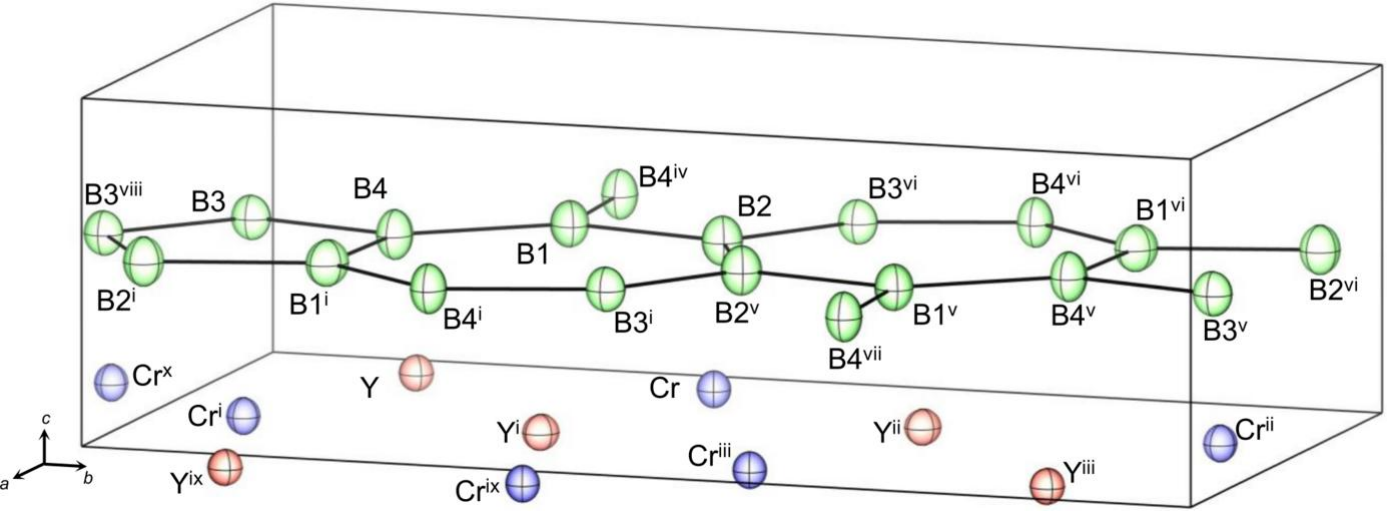
Displacement ellipsoids of each atom in YCrB_4_. Displacement ellipsoids are drawn at the 99% probability level. [Symmetry codes: (i) *x* + 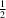 , −*y* + 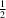 , *z*; (ii) −*x* + 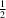 , *y* + 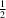 , −*z*; (iii) 1 − *x*, 1 − *y*, −*z*; (iv) *x* − 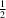 , −*y* + 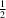 , *z*; (v) 1 − *x*, 1 − *y*, 1 − *z*; (vi) −*x* + 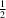 , *y* + 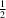 , 1 − *z*; (vii) −*x* + 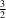 , *y* + 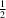 , 1 - *z;* (viii) 1 − *x*, −*y*, 1 − *z*; (ix) 1 + *x*, *y*, *z*; (*x*) −*x* + 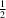 , *y* − 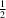 , −*z*].

A covalently bonded boron network in boride compounds plays an important role for thermal conductivity and mechanical and lattice dynamical properties. The Debye temperature *θ*_D_ was used to characterize these physical properties. Previous studies on inter­metallic boride compounds have also proposed that the bulk *θ*_D_ is associated with the rigidity of the boron network (Korsukova *et al.*, 1987 ▸; Levchenko *et al.*, 2006 ▸; Singh *et al.*, 2010 ▸). Using the isotropic atomic displacement parameter *U*_iso_ and Debye approximation (Willis & Pryor, 1975 ▸), the *θ*_D_ were derived using the following equation: <*U*_iso_^2^> = (3*h* ^2^ *T*) /(4π^2^ *m k*_B_ *θ*_D_^2^), where *h* is Planck’s constant, *m* is the mass of the atom, and *k*_B_ is the Boltzmann constant. The mean square <*U*_iso_^2^> for B atoms was calculated using the average *U*_iso_ for the boron sites. The anisotropic displacement parameters (ADPs) for each atom are listed in Table 2 ▸, with no significant anisotropy being observed in the ADPs of any atom (Fig. 3 ▸). The estimated *θ*_D_ for Y, Cr, and B were 413 (2), 524 (3), and 996 (25) K, respectively. Candan *et al.* (2019 ▸) studied the lattice-dynamical properties of YCrB_4_ using density functional theory and gave a calculated *θ*_D_ of 965 K that corresponds well with our estimated *θ*_D_ for the B atoms. This result indicates that the bulk *θ*_D_ of the AlB_2_-type-analogous compounds can be estimated from the ADPs for the B atom.

## 3. Synthesis and crystallization

The starting materials were Y (99.9%), Cr (99.95%), and B (99.5%). They were weighed in an atomic ratio Y:Cr:B = 1:1:4. The mixture was melted in an argon-arc melting furnace (ACM-01, Diavac). The product was then turned over and remelted three times to improve its chemical homogeneity. Homogeneous YCrB_4_ crystals were obtained.

## 4. Refinement details

Refinement was conducted using a space group of type *Pbam*, as reported by Kuz’ma, 1970 ▸. A correction for isotropic extinction was applied during the least-squares refinement. Final refinements were performed with inclusion of anisotropic ADPs to each atom. The final refinement results are listed in Table 3 ▸. The refinement was successful, with the *R* factor converging without any problems and no noticeable residuals.

## Supplementary Material

Crystal structure: contains datablock(s) I. DOI: 10.1107/S2056989023008952/pk2696sup1.cif

Structure factors: contains datablock(s) I. DOI: 10.1107/S2056989023008952/pk2696Isup2.hkl

CCDC reference: 2300781

Additional supporting information:  crystallographic information; 3D view; checkCIF report

## Figures and Tables

**Table 1:** Selected bond lengths (Å) in YCrB_4_

*CrB_10_ Penta­gonal prism*		*Five-membered ring*	
Cr—B3×2	2.2677 (15)	B3–B3^viii^	1.724 (4)
Cr—B3×2	2.2723 (9)	B2^i^–B3^viii^	1.741 (4)
Cr—B1×2	2.2962 (11)	B3–B4	1.742 (2)
Cr—B2×2	2.3081 (16)	B1^i^–B2^i^	1.807 (2)
Cr—B4×6	2.3254 (10)	B1^i^–B4	1.828 (2)
			
*YB_14_ Heptagonal prism*		*Seven-membered ring*	
Y—B3×2	2.6177 (16)	B2^v^–B3^i^	1.741 (3)
Y—B4×2	2.6582 (11)	B3^i^–B4^i^	1.742 (2)
Y—B2×2	2.6959 (15)	B2–B2^v^	1.788 (4)
Y—B1×2	2.7003 (12)	B1–B2	1.807 (2)
Y—B2×6	2.7241 (11)	B1^i^–B4	1.828 (2)
Y—B1×2	2.7350 (13)	B1–B4	1.832 (2)
Y—B4×6	2.7478 (14)	B1^i^–B4^i^	1.832 (2)
			
*Inter­planar atomic distances*			
Cr^i^—Cr^*x*^	2.3745 (4)	Y^i^—Y	3.7446 (3)
Y^i^—Cr^iii^	3.0517 (4)	Y^i^—Y^ix^	3.7446 (3)
Y^i^—Cr^ix^	3.0760 (3)	Y^i^—Y^ii^	3.7653 (5)
Y^i^—Cr	3.0789 (3)		
Y^i^—Cr^i^	3.0803 (4)		

Symmetry codes: (i) *x* + 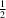 , −*y* + 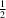 , *z*; (ii) −*x* + 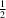 , *y* + 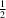 , −*z*; (iii) 1 − *x*, 1 − *y*, −*z*; (v) 1 − *x*, 1 − *y*, 1 − *z*; (viii) 1 − *x*, −*y*, 1 − *z*; (ix) 1 + *x*, *y*, *z*; (*x*) −*x* + 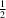 , *y* − 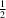 , −*z*]

**Table 2:** Atomic coordinates and anisotropic displacement parameters (10 ^3^Å^2^) for YCrB_4_ The Y and Cr atoms lie on the Wyckoff sites 4*h* (*x*, *y*, 0), and the B atoms occupy the 4*g* (*x*, *y*, 1/2) site. The anisotropic displacement factor exponent takes the form: −2π^2^[(ha*)^2^ *U*_11_ + ⋯ + 2hka*b*U_12_]. *U*_iso_ is defined as a third of the trace of the orthogonalized *U*_*ij*_ tensor. *U*_12_ = *U*_23_ = 0.

Atom	*x*	*y*	*U*_11_	*U*_22_	*U*_33_	*U*_12_	*U*_iso_
Y	0.12446 (2)	0.15077 (2)	0.00298 (4)	0.00275 (4)	0.00284 (4)	0.00027 (4)	0.00285 (3)
Cr	0.12421 (4)	0.41902 (2)	0.00262 (6)	0.00241 (6)	0.00297 (6)	0.00007 (6)	0.00267 (3)
B1	0.2818 (3)	0.31614 (15)	0.0045 (5)	0.0035 (4)	0.0047 (5)	0.0007 (4)	0.0042 (2)
B2	0.3630 (4)	0.46779 (13)	0.0037 (6)	0.0035 (4)	0.0053 (5)	0.0001 (5)	0.0042 (2)
B3	0.3869 (4)	0.04697 (12)	0.0029 (5)	0.0034 (4)	0.0043 (4)	−0.0001 (5)	0.0036 (2)
B4	0.4746 (3)	0.19170 (13)	0.0041 (5)	0.0028 (5)	0.0054 (5)	−0.0001 (4)	0.0041 (2)

**Table 3:** Experimental details

Crystal data	
Chemical formula	YCrB_4_
*M*_r_	184.15
Crystal system, space group	Orthorhombic, *P* *b* *a* *m*
Temperature (K)	294
*a*, *b*, *c* (Å)	5.9425 (2), 11.4831 (4), 3.46425 (12)
*V* (Å^3^)	236.40 (1)
*Z*	4
Radiation type	Mo *K*α
μ (mm^−1^)	28.57
Crystal size (mm)	0.05 × 0.03 × 0.03

Data collection	
Diffractometer	XtaLAB Synergy, HyPix
Absorption correction	Numerical (*CrysAlis PRO*; Rigaku OD, 2021 ▸)
*T*_min_, *T*_max_	0.367, 0.655
No. of measured, independent and observed [*I* > 2σ(*I*)] reflections	13376, 1074, 865
*R*_int_	0.036
(sin θ/λ)_max_ (Å^−1^)	0.992

Refinement	
*R*[*F* ^2^ > 2σ(*F* ^2^)], *wR*(*F* ^2^), *S*	0.015, 0.029, 1.11
No. of reflections	1074
No. of parameters	38
Δρ_max_, Δρ_min_ (e Å^−3^)	0.58, −0.71

Computer programs: *CrysAlis PRO* (Rigaku OD, 2021 ▸), *SHELXT* (Sheldrick, 2015*a* ▸), *SHELXL* (Sheldrick, 2015*b* ▸), and *VESTA* (Momma & Izumi, 2011 ▸).

## supplementary crystallographic information

### Crystal data

**Table:**

YCrB_4_	*D*_x_ = 5.174 Mg m^−^^3^
*M**_r_* = 184.15	Mo *K*α radiation, λ = 0.71073 Å
Orthorhombic, *P**b**a**m*	Cell parameters from 6090 reflections
*a* = 5.9425 (2) Å	θ = 3.5–44.9°
*b* = 11.4831 (4) Å	µ = 28.57 mm^−^^1^
*c* = 3.46425 (12) Å	*T* = 294 K
*V* = 236.40 (1) Å^3^	Block, metallic
*Z* = 4	0.05 × 0.03 × 0.03 mm
*F*(000) = 332	

### Data collection

**Table:**

XtaLAB Synergy, HyPix diffractometer	1074 independent reflections
Radiation source: micro-focus sealed X-ray tube, PhotonJet (Mo) X-ray Source	865 reflections with *I* > 2σ(*I*)
Mirror monochromator	*R*_int_ = 0.036
Detector resolution: 10.0000 pixels mm^-1^	θ_max_ = 44.9°, θ_min_ = 3.6°
ω scans	*h* = −11→11
Absorption correction: numerical (CrysAlisPro; Rigaku OD, 2021)	*k* = −22→22
*T*_min_ = 0.367, *T*_max_ = 0.655	*l* = −6→6
13376 measured reflections	

### Refinement

**Table:**

Refinement on *F*^2^	0 restraints
Least-squares matrix: full	*w* = 1/[σ^2^(*F*_o_^2^) + (0.0074*P*)^2^ + 0.1807*P*] where *P* = (*F*_o_^2^ + 2*F*_c_^2^)/3
*R*[*F*^2^ > 2σ(*F*^2^)] = 0.015	(Δ/σ)_max_ = 0.020
*wR*(*F*^2^) = 0.029	Δρ_max_ = 0.58 e Å^−^^3^
*S* = 1.11	Δρ_min_ = −0.71 e Å^−^^3^
1074 reflections	Extinction correction: *SHELXL* (Sheldrick, 2015b), Fc^*^=kFc[1+0.001xFc^2^λ^3^/sin(2θ)]^-1/4^
38 parameters	Extinction coefficient: 0.0111 (7)

### Special details

**Table:**

Geometry. All esds (except the esd in the dihedral angle between two l.s. planes) are estimated using the full covariance matrix. The cell esds are taken into account individually in the estimation of esds in distances, angles and torsion angles; correlations between esds in cell parameters are only used when they are defined by crystal symmetry. An approximate (isotropic) treatment of cell esds is used for estimating esds involving l.s. planes.

### Fractional atomic coordinates and isotropic or equivalent isotropic displacement parameters (Å^2^)

**Table:**

	*x*	*y*	*z*	*U*_iso_*/*U*_eq_	
Y	0.12446 (2)	0.15077 (2)	0.000000	0.00285 (3)	
Cr	0.12421 (4)	0.41902 (2)	0.000000	0.00267 (3)	
B1	0.2818 (3)	0.31614 (15)	0.500000	0.0042 (2)	
B2	0.3630 (4)	0.46779 (13)	0.500000	0.0042 (2)	
B3	0.3869 (4)	0.04697 (12)	0.500000	0.0036 (2)	
B4	0.4746 (3)	0.19170 (13)	0.500000	0.0041 (2)	

### Atomic displacement parameters (Å^2^)

**Table:**

	*U*^11^	*U*^22^	*U*^33^	*U*^12^	*U*^13^	*U*^23^
Y	0.00298 (4)	0.00275 (4)	0.00284 (4)	0.00027 (4)	0.000	0.000
Cr	0.00262 (6)	0.00241 (6)	0.00297 (6)	0.00007 (6)	0.000	0.000
B1	0.0045 (5)	0.0035 (4)	0.0047 (5)	0.0007 (4)	0.000	0.000
B2	0.0037 (6)	0.0035 (4)	0.0053 (5)	0.0001 (5)	0.000	0.000
B3	0.0029 (5)	0.0034 (4)	0.0043 (4)	0.0000 (5)	0.000	0.000
B4	0.0041 (5)	0.0028 (5)	0.0054 (5)	−0.0001 (4)	0.000	0.000

### Geometric parameters (Å, º)

**Table:**

Y—B3	2.6177 (16)	Cr—B3^vii^	2.2723 (9)
Y—B3^i^	2.6177 (16)	Cr—B1^i^	2.2962 (11)
Y—B4^ii^	2.6582 (11)	Cr—B1	2.2962 (11)
Y—B4^iii^	2.6582 (11)	Cr—B2^i^	2.3081 (16)
Y—B2^iii^	2.6959 (15)	Cr—B2	2.3081 (16)
Y—B2^ii^	2.6959 (15)	Cr—B4^iii^	2.3254 (10)
Y—B1^iii^	2.7003 (12)	Cr—B4^ii^	2.3254 (10)
Y—B1^ii^	2.7003 (12)	Cr—Cr^viii^	2.3745 (4)
Y—B2^iv^	2.7241 (11)	B1—B2	1.807 (2)
Y—B2^v^	2.7241 (11)	B1—B4^ii^	1.828 (2)
Y—B1	2.7350 (13)	B1—B4	1.832 (2)
Y—B1^i^	2.7350 (13)	B2—B3^vi^	1.741 (4)
Cr—B3^iii^	2.2677 (15)	B2—B2^ix^	1.789 (5)
Cr—B3^ii^	2.2677 (15)	B3—B3^x^	1.724 (4)
Cr—B3^vi^	2.2723 (9)	B3—B4	1.742 (2)
			
B3—Y—B3^i^	82.86 (6)	B2—B1—Cr^xi^	67.24 (6)
B3—Y—B4^ii^	94.49 (3)	B4^ii^—B1—Cr^xi^	67.55 (5)
B3^i^—Y—B4^ii^	160.16 (5)	B4—B1—Cr^xi^	131.03 (3)
B3—Y—B4^iii^	160.16 (5)	Cr—B1—Cr^xi^	97.93 (6)
B3^i^—Y—B4^iii^	94.49 (3)	B2—B1—Y^xii^	70.31 (7)
B4^ii^—Y—B4^iii^	81.33 (4)	B4^ii^—B1—Y^xii^	139.48 (3)
B3—Y—B2^iii^	122.58 (4)	B4—B1—Y^xii^	68.78 (5)
B3^i^—Y—B2^iii^	71.84 (4)	Cr—B1—Y^xii^	135.97 (7)
B4^ii^—Y—B2^iii^	124.69 (6)	Cr^xi^—B1—Y^xii^	75.60 (2)
B4^iii^—Y—B2^iii^	74.45 (4)	B2—B1—Y^xiii^	70.31 (7)
B3—Y—B2^ii^	71.84 (4)	B4^ii^—B1—Y^xiii^	139.48 (3)
B3^i^—Y—B2^ii^	122.58 (4)	B4—B1—Y^xiii^	68.78 (5)
B4^ii^—Y—B2^ii^	74.45 (4)	Cr—B1—Y^xiii^	75.60 (2)
B4^iii^—Y—B2^ii^	124.69 (5)	Cr^xi^—B1—Y^xiii^	135.97 (7)
B2^iii^—Y—B2^ii^	79.96 (5)	Y^xii^—B1—Y^xiii^	79.80 (4)
B3—Y—B1^iii^	159.68 (5)	B2—B1—Y^xi^	139.50 (3)
B3^i^—Y—B1^iii^	95.10 (4)	B4^ii^—B1—Y^xi^	67.94 (6)
B4^ii^—Y—B1^iii^	93.99 (4)	B4—B1—Y^xi^	70.86 (5)
B4^iii^—Y—B1^iii^	39.96 (4)	Cr—B1—Y^xi^	134.07 (6)
B2^iii^—Y—B1^iii^	39.13 (5)	Cr^xi^—B1—Y^xi^	74.94 (2)
B2^ii^—Y—B1^iii^	92.78 (5)	Y^xii^—B1—Y^xi^	87.09 (2)
B3—Y—B1^ii^	95.10 (4)	Y^xiii^—B1—Y^xi^	139.62 (6)
B3^i^—Y—B1^ii^	159.68 (5)	B2—B1—Y	139.50 (3)
B4^ii^—Y—B1^ii^	39.96 (4)	B4^ii^—B1—Y	67.94 (6)
B4^iii^—Y—B1^ii^	93.99 (4)	B4—B1—Y	70.86 (5)
B2^iii^—Y—B1^ii^	92.78 (5)	Cr—B1—Y	74.94 (2)
B2^ii^—Y—B1^ii^	39.13 (5)	Cr^xi^—B1—Y	134.07 (6)
B1^iii^—Y—B1^ii^	79.80 (4)	Y^xii^—B1—Y	139.62 (6)
B3—Y—B2^iv^	93.05 (4)	Y^xiii^—B1—Y	87.09 (2)
B3^i^—Y—B2^iv^	37.98 (7)	Y^xi^—B1—Y	78.59 (4)
B4^ii^—Y—B2^iv^	161.52 (6)	B3^vi^—B2—B2^ix^	124.09 (14)
B4^iii^—Y—B2^iv^	96.88 (3)	B3^vi^—B2—B1	105.99 (17)
B2^iii^—Y—B2^iv^	38.53 (9)	B2^ix^—B2—B1	129.92 (17)
B2^ii^—Y—B2^iv^	92.00 (3)	B3^vi^—B2—Cr	66.58 (9)
B1^iii^—Y—B2^iv^	73.82 (5)	B2^ix^—B2—Cr	131.30 (4)
B1^ii^—Y—B2^iv^	122.48 (5)	B1—B2—Cr	66.55 (6)
B3—Y—B2^v^	37.98 (7)	B3^vi^—B2—Cr^xi^	66.58 (9)
B3^i^—Y—B2^v^	93.05 (4)	B2^ix^—B2—Cr^xi^	131.30 (4)
B4^ii^—Y—B2^v^	96.88 (3)	B1—B2—Cr^xi^	66.55 (6)
B4^iii^—Y—B2^v^	161.52 (6)	Cr—B2—Cr^xi^	97.26 (9)
B2^iii^—Y—B2^v^	92.00 (3)	B3^vi^—B2—Y^xii^	139.05 (5)
B2^ii^—Y—B2^v^	38.53 (9)	B2^ix^—B2—Y^xii^	71.58 (9)
B1^iii^—Y—B2^v^	122.48 (5)	B1—B2—Y^xii^	70.56 (6)
B1^ii^—Y—B2^v^	73.82 (5)	Cr—B2—Y^xii^	135.56 (6)
B2^iv^—Y—B2^v^	78.97 (4)	Cr^xi^—B2—Y^xii^	75.50 (2)
B3—Y—B1	72.15 (4)	B3^vi^—B2—Y^xiii^	139.05 (5)
B3^i^—Y—B1	122.11 (5)	B2^ix^—B2—Y^xiii^	71.58 (9)
B4^ii^—Y—B1	39.59 (4)	B1—B2—Y^xiii^	70.56 (6)
B4^iii^—Y—B1	93.14 (3)	Cr—B2—Y^xiii^	75.50 (2)
B2^iii^—Y—B1	162.65 (5)	Cr^xi^—B2—Y^xiii^	135.56 (6)
B2^ii^—Y—B1	98.09 (3)	Y^xii^—B2—Y^xiii^	79.95 (5)
B1^iii^—Y—B1	124.49 (3)	B3^vi^—B2—Y^vi^	67.70 (5)
B1^ii^—Y—B1	75.75 (3)	B2^ix^—B2—Y^vi^	69.88 (7)
B2^iv^—Y—B1	158.26 (6)	B1—B2—Y^vi^	138.64 (4)
B2^v^—Y—B1	97.09 (4)	Cr—B2—Y^vi^	132.94 (8)
B3—Y—B1^i^	122.11 (5)	Cr^xi^—B2—Y^vi^	74.15 (3)
B3^i^—Y—B1^i^	72.15 (4)	Y^xii^—B2—Y^vi^	88.01 (3)
B4^ii^—Y—B1^i^	93.14 (3)	Y^xiii^—B2—Y^vi^	141.47 (9)
B4^iii^—Y—B1^i^	39.59 (4)	B3^vi^—B2—Y^vii^	67.70 (5)
B2^iii^—Y—B1^i^	98.09 (3)	B2^ix^—B2—Y^vii^	69.88 (7)
B2^ii^—Y—B1^i^	162.65 (5)	B1—B2—Y^vii^	138.64 (4)
B1^iii^—Y—B1^i^	75.75 (3)	Cr—B2—Y^vii^	74.15 (3)
B1^ii^—Y—B1^i^	124.49 (3)	Cr^xi^—B2—Y^vii^	132.94 (8)
B2^iv^—Y—B1^i^	97.09 (4)	Y^xii^—B2—Y^vii^	141.47 (9)
B2^v^—Y—B1^i^	158.26 (6)	Y^xiii^—B2—Y^vii^	88.01 (3)
B1—Y—B1^i^	78.59 (4)	Y^vi^—B2—Y^vii^	78.97 (4)
B3^iii^—Cr—B3^ii^	99.61 (9)	B3^x^—B3—B2^v^	109.78 (13)
B3^iii^—Cr—B3^vi^	116.93 (3)	B3^x^—B3—B4	111.32 (18)
B3^ii^—Cr—B3^vi^	44.63 (10)	B2^v^—B3—B4	138.90 (18)
B3^iii^—Cr—B3^vii^	44.63 (10)	B3^x^—B3—Cr^xii^	67.83 (9)
B3^ii^—Cr—B3^vii^	116.93 (3)	B2^v^—B3—Cr^xii^	128.34 (4)
B3^vi^—Cr—B3^vii^	99.33 (5)	B4—B3—Cr^xii^	69.48 (6)
B3^iii^—Cr—B1^i^	76.47 (5)	B3^x^—B3—Cr^xiii^	67.83 (9)
B3^ii^—Cr—B1^i^	156.70 (6)	B2^v^—B3—Cr^xiii^	128.34 (4)
B3^vi^—Cr—B1^i^	156.85 (7)	B4—B3—Cr^xiii^	69.48 (6)
B3^vii^—Cr—B1^i^	76.67 (5)	Cr^xii^—B3—Cr^xiii^	99.61 (9)
B3^iii^—Cr—B1	156.70 (6)	B3^x^—B3—Cr^iv^	67.54 (6)
B3^ii^—Cr—B1	76.47 (5)	B2^v^—B3—Cr^iv^	68.75 (5)
B3^vi^—Cr—B1	76.67 (5)	B4—B3—Cr^iv^	128.73 (4)
B3^vii^—Cr—B1	156.85 (7)	Cr^xii^—B3—Cr^iv^	135.37 (10)
B1^i^—Cr—B1	97.94 (6)	Cr^xiii^—B3—Cr^iv^	63.07 (3)
B3^iii^—Cr—B2^i^	76.55 (4)	B3^x^—B3—Cr^v^	67.54 (6)
B3^ii^—Cr—B2^i^	156.04 (5)	B2^v^—B3—Cr^v^	68.75 (5)
B3^vi^—Cr—B2^i^	115.66 (5)	B4—B3—Cr^v^	128.73 (4)
B3^vii^—Cr—B2^i^	44.67 (8)	Cr^xii^—B3—Cr^v^	63.07 (3)
B1^i^—Cr—B2^i^	46.21 (5)	Cr^xiii^—B3—Cr^v^	135.37 (10)
B1—Cr—B2^i^	116.12 (6)	Cr^iv^—B3—Cr^v^	99.33 (5)
B3^iii^—Cr—B2	156.04 (5)	B3^x^—B3—Y	138.55 (3)
B3^ii^—Cr—B2	76.55 (4)	B2^v^—B3—Y	74.33 (10)
B3^vi^—Cr—B2	44.67 (8)	B4—B3—Y	75.16 (6)
B3^vii^—Cr—B2	115.66 (5)	Cr^xii^—B3—Y	142.90 (6)
B1^i^—Cr—B2	116.12 (6)	Cr^xiii^—B3—Y	77.682 (19)
B1—Cr—B2	46.22 (5)	Cr^iv^—B3—Y	76.87 (3)
B2^i^—Cr—B2	97.26 (9)	Cr^v^—B3—Y	141.41 (10)
B3^iii^—Cr—B4^iii^	44.55 (5)	B3^x^—B3—Y^xi^	138.55 (3)
B3^ii^—Cr—B4^iii^	115.17 (6)	B2^v^—B3—Y^xi^	74.33 (10)
B3^vi^—Cr—B4^iii^	155.54 (7)	B4—B3—Y^xi^	75.15 (6)
B3^vii^—Cr—B4^iii^	76.98 (5)	Cr^xii^—B3—Y^xi^	77.682 (19)
B1^i^—Cr—B4^iii^	46.59 (5)	Cr^xiii^—B3—Y^xi^	142.89 (6)
B1—Cr—B4^iii^	115.88 (4)	Cr^iv^—B3—Y^xi^	141.41 (10)
B2^i^—Cr—B4^iii^	78.97 (5)	Cr^v^—B3—Y^xi^	76.87 (3)
B2—Cr—B4^iii^	157.93 (6)	Y—B3—Y^xi^	82.86 (6)
B3^iii^—Cr—B4^ii^	115.17 (6)	B3—B4—B1^xiii^	104.59 (12)
B3^ii^—Cr—B4^ii^	44.55 (5)	B3—B4—B1	123.87 (12)
B3^vi^—Cr—B4^ii^	76.98 (5)	B1^xiii^—B4—B1	131.54 (10)
B3^vii^—Cr—B4^ii^	155.54 (7)	B3—B4—Cr^xiii^	65.97 (6)
B1^i^—Cr—B4^ii^	115.88 (4)	B1^xiii^—B4—Cr^xiii^	65.87 (5)
B1—Cr—B4^ii^	46.59 (5)	B1—B4—Cr^xiii^	131.74 (3)
B2^i^—Cr—B4^ii^	157.93 (6)	B3—B4—Cr^xii^	65.97 (6)
B2—Cr—B4^ii^	78.97 (5)	B1^xiii^—B4—Cr^xii^	65.87 (5)
B4^iii^—Cr—B4^ii^	96.30 (6)	B1—B4—Cr^xii^	131.74 (3)
B3^iii^—Cr—Cr^viii^	58.56 (3)	Cr^xiii^—B4—Cr^xii^	96.30 (6)
B3^ii^—Cr—Cr^viii^	58.56 (3)	B3—B4—Y^xii^	138.56 (3)
B3^vi^—Cr—Cr^viii^	58.37 (4)	B1^xiii^—B4—Y^xii^	72.47 (6)
B3^vii^—Cr—Cr^viii^	58.37 (4)	B1—B4—Y^xii^	71.26 (5)
B1^i^—Cr—Cr^viii^	131.03 (3)	Cr^xiii^—B4—Y^xii^	136.83 (7)
B1—Cr—Cr^viii^	131.03 (3)	Cr^xii^—B4—Y^xii^	76.03 (2)
B2^i^—Cr—Cr^viii^	101.08 (4)	B3—B4—Y^xiii^	138.56 (3)
B2—Cr—Cr^viii^	101.08 (4)	B1^xiii^—B4—Y^xiii^	72.47 (6)
B4^iii^—Cr—Cr^viii^	100.98 (4)	B1—B4—Y^xiii^	71.26 (5)
B4^ii^—Cr—Cr^viii^	100.98 (4)	Cr^xiii^—B4—Y^xiii^	76.03 (2)
B3^iii^—Cr—Y^vii^	98.87 (4)	Cr^xii^—B4—Y^xiii^	136.83 (7)
B3^ii^—Cr—Y^vii^	98.87 (4)	Y^xii^—B4—Y^xiii^	81.33 (4)
B3^vi^—Cr—Y^vii^	56.65 (4)	B3—B4—Y^xi^	67.06 (7)
B3^vii^—Cr—Y^vii^	56.65 (4)	B1^xiii^—B4—Y^xi^	138.40 (4)
B1^i^—Cr—Y^vii^	104.42 (4)	B1—B4—Y^xi^	70.11 (5)
B1—Cr—Y^vii^	104.42 (4)	Cr^xiii^—B4—Y^xi^	131.73 (6)
B2^i^—Cr—Y^vii^	59.17 (4)	Cr^xii^—B4—Y^xi^	74.13 (2)
B2—Cr—Y^vii^	59.17 (4)	Y^xii^—B4—Y^xi^	87.67 (2)
B4^iii^—Cr—Y^vii^	131.60 (3)	Y^xiii^—B4—Y^xi^	141.34 (6)
B4^ii^—Cr—Y^vii^	131.60 (3)	B3—B4—Y	67.06 (7)
Cr^viii^—Cr—Y^vii^	67.744 (10)	B1^xiii^—B4—Y	138.40 (4)
B2—B1—B4^ii^	108.31 (12)	B1—B4—Y	70.11 (5)
B2—B1—B4	125.79 (12)	Cr^xiii^—B4—Y	74.13 (2)
B4^ii^—B1—B4	125.90 (10)	Cr^xii^—B4—Y	131.73 (6)
B2—B1—Cr	67.24 (6)	Y^xii^—B4—Y	141.34 (6)
B4^ii^—B1—Cr	67.55 (5)	Y^xiii^—B4—Y	87.67 (2)
B4—B1—Cr	131.03 (3)	Y^xi^—B4—Y	78.16 (4)

Symmetry codes: (i) *x*, *y*, *z*−1; (ii) *x*−1/2, −*y*+1/2, *z*; (iii) *x*−1/2, −*y*+1/2, *z*−1; (iv) −*x*+1/2, *y*−1/2, −*z*; (v) −*x*+1/2, *y*−1/2, −*z*+1; (vi) −*x*+1/2, *y*+1/2, −*z*+1; (vii) −*x*+1/2, *y*+1/2, −*z*; (viii) −*x*, −*y*+1, −*z*; (ix) −*x*+1, −*y*+1, −*z*+1; (x) −*x*+1, −*y*, −*z*+1; (xi) *x*, *y*, *z*+1; (xii) *x*+1/2, −*y*+1/2, *z*+1; (xiii) *x*+1/2, −*y*+1/2, *z*.
